# Interleukin-38 overexpression in keratinocytes limits desquamation but does not affect the global severity of imiquimod-induced skin inflammation in mice

**DOI:** 10.3389/fimmu.2024.1387921

**Published:** 2024-07-25

**Authors:** Arnaud Huard, Emiliana Rodriguez, Dominique Talabot-Ayer, Andreas Weigert, Gaby Palmer

**Affiliations:** ^1^ Division of Rheumatology, Department of Medicine, Faculty of Medicine, University of Geneva, Geneva, Switzerland; ^2^ Department of Pathology and Immunology, Faculty of Medicine, University of Geneva, Geneva, Switzerland; ^3^ Geneva Centre for Inflammation Research, Geneva, Switzerland; ^4^ Institute of Biochemistry I, Faculty of Medicine, Goethe-University Frankfurt, Frankfurt, Germany

**Keywords:** cytokines, epidermis, inflammation, interleukin-38, psoriasis

## Abstract

Psoriasis is a common chronic inflammatory skin disease that significantly impacts the patients’ quality of life. Recent studies highlighted the function of the interleukin (IL)-1 family member IL-38 in skin homeostasis and suggested an anti-inflammatory role for this cytokine in psoriasis. In this study, we generated mice specifically overexpressing the IL-38 protein in epidermal keratinocytes. We confirmed IL-38 overexpression in the skin by Western blotting. We further detected the protein by ELISA in the plasma, as well as in conditioned media of skin explants isolated from IL-38 overexpressing mice, indicating that IL-38 produced in the epidermis is released from keratinocytes and can be found in the circulation. Unexpectedly, epidermal IL-38 overexpression did not impact the global severity of imiquimod (IMQ)-induced skin inflammation, Similarly, keratinocyte activation and differentiation in IMQ-treated skin were not affected by increased IL-38 expression and there was no global effect on local or systemic inflammatory responses. Nevertheless, we observed a selective inhibition of CXCL1 and IL-6 production in response to IMQ in IL-38 overexpressing skin, as well as reduced *Ly6g* mRNA levels, suggesting decreased neutrophil infiltration. Epidermal IL-38 overexpression also selectively affected the desquamation process during IMQ-induced psoriasis, as illustrated by reduced plaque formation. Taken together, our results validate the generation of a new mouse line allowing for tissue-specific IL-38 overexpression. Interestingly, epidermal IL-38 overexpression selectively affected specific disease-associated readouts during IMQ-induced psoriasis, suggesting a more complex role of IL-38 in the inflamed skin than previously recognized. In particular, our data highlight a potential involvement of IL-38 in the regulation of skin desquamation.

## Introduction

Psoriasis is a common chronic inflammatory skin disease that significantly impacts the patients’ quality of life. Psoriatic skin inflammation is characterized by the formation of demarcated erythematous plaques, covered by lamellar scales (1). The pathogenesis of psoriasis remains incompletely understood and, despite impressive progress made in its treatment, this disease still presents therapeutic challenges (2).

Recent studies highlighted the role of interleukin (IL)-38 in skin homeostasis and inflammation. IL-38 is a member of the IL-1 family of cytokines and shares over 40% homology with the anti-inflammatory IL-1 family members IL-1 receptor antagonist (IL-1Ra) and IL-36 receptor antagonist (IL-36Ra) (3). IL-38 is produced by immune cells, particularly macrophages and B cells (4–6), but its highest constitutive expression is observed in the skin, where epidermal keratinocytes are a major producing cell type (7–9).

IL-38 accumulates in the cytoplasm of keratinocytes in healthy human epidermis and was proposed to promote keratinocyte differentiation (9). These data corroborate observations showing decreased keratinocyte differentiation during imiquimod (IMQ)-induced psoriasis in IL-38 deficient mice (7). The effects of IL-38 on keratinocyte proliferation are less clear. Indeed, while IL-38 overexpression reduced the proliferation of a human keratinocyte cell line *in vitro* (8), a recent study showed that specifically knocking out IL-38 in mouse epidermal keratinocytes downregulated their proliferation in a skin cancer model (10). In addition, both *in vitro* data and mouse studies suggested an anti-inflammatory role for IL-38 in psoriasis (7, 11). IL-38 prevented the induction of pro-inflammatory mediators by IL-36γ in cultured human keratinocytes and dermal vascular endothelial cells, and injection of recombinant IL-38 protein reduced the severity of IMQ-induced skin inflammation in mice (11). In line with these findings, mice that were totally deficient in IL-38 showed a delayed resolution of IMQ-induced skin inflammation, associated with increased IL-17A production from γδ T cells (7).

In addition to total or tissue-specific IL-38 KO or injection of recombinant IL-38 protein, several groups previously used IL-38 overexpression to study the effects of this cytokine *in vivo* (reviewed in (12)). The strategies used included injection of adeno-associated viruses, lentiviruses or plasmids, which do not allow for cell-type specific overexpression and may lead to variable overexpression levels (13–15). In the present study, we generated a floxed mouse line that allows for conditional, Cre-dependent overexpression of IL-38 from the ROSA locus. As a proof of concept, we crossed this strain to mice producing the Cre recombinase in epidermal keratinocytes under the control of the keratin (*Krt*)5 promoter, which led to IL-38 overexpression specifically in the skin. To our knowledge, this is the first genetically modified mouse line showing tissue-specific overexpression of IL-38. We then used these mice in an IMQ-induced psoriasis model and observed that IL-38 overexpression in the epidermis attenuated scaling, but did not impact the global severity of IMQ-induced skin inflammation.

## Materials and methods

### Mice

The B6-Gt(ROSA)26Sor^tm1(Il1f10)iTL^ (ILFO) mouse strain (**Supplementary Figure S1**) was generated by Ingenious targeting laboratory (iTL; Ronkonkoma, NY, USA). Briefly, an expression cassette containing an *Il1f10* cDNA encoding the full length mouse IL-38 protein (mouse_Il1f10-201_ENSMUST00000058056_CodingSequence_152aa) was inserted by homologous recombination into the Gt(ROSA)26S (ROSA) locus in iTL C57Bl/6N (IN2) embryonic stem cells. Correctly targeted IN2 embryonic stem cells were then used to generate C57BL/6N ILFO mice. The targeting vector was designed so that the overexpression of mouse IL-38 is driven by a strong ubiquitous pCAG promoter. However, transcription of the transgene encoding IL-38 is blocked in ILFO mice by the presence of a LoxP flanked Neo-STOP cassette inserted between the pCAG promoter and the *Il1f10* transcription start site. Thus, in ILFO mice, the inserted *Il1f10* transgene is expected to be silent and overexpression of the IL-38 protein should only be observed after excision of the STOP cassette by Cre recombinase, upon crossbreeding of ILFO mice with Cre expressing mouse lines (**Supplementary Figure S1B**). In order to overexpress IL-38 in the epidermis, homozygous ILFO knock in (KI) females were mated with hemizygous K5-Cre transgenic (tg) males (kindly provided by Dr. Vincent Flacher, Institute of Cellular and Molecular Biology, Strasbourg), which express Cre recombinase under the control of the human *Krt5* promoter. These breedings generated K5-Cre tg ILFO^+/ki^ (K5-ILFO) mice and their ILFO^+/ki^ littermates that were used as controls. IL-38KO mice (7) were backcrossed to the C57BL/6N background to >99% purity, as assessed using a 384 single-nucleotide polymorphism (SNP) panel with SNPs spread across the genome at 7 Mbp intervals (Charles River Laboratories, Wilmington, MA). All mice were bred and maintained in the conventional area of the animal facility at the Geneva University Faculty of Medicine. Animal studies were approved by the Geneva Veterinarian Office (authorization GE161) and complied with the requirements defined by the Swiss regulation (federal animal protection ordinances and law).

### Imiquimod-induced skin inflammation

Imiquimod (IMQ)-induced skin inflammation was induced in male and female K5-ILFO mice and in their control ILFO littermates, as previously described (van der Fits et al., 2009). Briefly, the back skin of 8-12 week-old mice was shaved one day before starting the experiment. 62.5 mg of Aldara cream, containing 5% IMQ (3M Pharmaceuticals, Maplewood, MN, USA) were then applied daily on the back skin for 3 or 7 consecutive days. The severity of skin inflammation was evaluated according to a cumulative Psoriasis Area Severity Index (PASI) scoring system based on the extent of skin redness, thickness and scaling (16). Mice were sacrificed and skin, lung and plasma samples were collected on day 3 or day 7 for further analyses.

### Western blotting

Skin and lung samples of naïve and IMQ-treated mice were lysed in 500 μl TN buffer (50 mM Tris, 150 mM NaCl, complete EDTA-free protease inhibitor mixture; Roche Diagnostics, Rotkreuz, Switzerland). Samples were cleared by centrifugation, and total protein content was determined with the DC protein assay kit (Bio-Rad Laboratories, Cressier, Switzerland). HEK 293T cells were transiently transfected with an expression vector encoding full-length mouse IL-38 (GenBank accession NM_153077.3) using the jetPRIME reagent (Polyplus-transfection, Illkirch, France). IL-38 overexpressing HEK cells were then lysed 24h after transfection in RIPA buffer (Sigma-Aldrich, Darmstadt, Germany) to be used as a positive control. Skin and lung lysates of IL-38KO mice were used as a negative control to validate the specificity of IL-38 protein detection. Skin, lung and HEK cell lysates were separated by electrophoresis on 4–12% gradient polyacrylamide gels (Invitrogen, Waltham, MA, USA) and transferred onto PVDF membranes (Macherey-Nagel, Oensingen, Switzerland). Membranes were blocked in 4% non-fat milk and incubated with rabbit anti-IL-38 (1/500, ab180898, Abcam, Cambridge, UK) and mouse anti-GAPDH (1/2000, MAB374, Millipore, Darmstadt, Germany) antibodies in 2% non-fat milk buffer. Bands were visualized using appropriate IRDye 800- and IRDye 680-coupled secondary antibodies (1/10000) and the Li-Cor Odyssey imaging system (LICOR Biosciences, Bad Homburg, Germany), and quantified using the Empiria studio 3.0 software (LICOR Biosciences).

### ELISA

For skin explant cultures, mice were sacrificed and the whole back skin was shaved and collected. Four pieces of skin were then excised using a circular punch biopsy tool to generate full thickness skin samples of identical surface area. Each explant (diameter: 5 mm) was incubated in 200 μl assay medium (DMEM, 5% FCS, penicillin/streptomycin) at 37°C, as described (17) and conditioned media were harvested after 24h. IL-38 levels in conditioned media and in plasma were assessed by ELISA (R&D Systems, Abingdon, UK) following the manufacturer’s instructions. For skin explants, the mean of the results obtained for the 4 individual punch biopsies, considered as technical replicates, was used for each mouse.

### Histology

Skin of naïve and IMQ-treated mice was fixed in buffered formalin and embedded in paraffin. Four-micrometer-thick sections were deparaffinized, rehydrated and stained using hematoxylin-eosin (H&E). Images were acquired on the Axioscan.Z1 widefield scanner (Carl Zeiss Microscopy, Feldbach, Switzerland). Epidermal thickness was measured using the Zen 3.4 software (Carl Zeiss Microscopy).

### RNA extraction and RT-PCR

Total RNA from mouse organs was extracted using TRIzol (Invitrogen) and RNeasy columns (Qiagen, Hilden, Germany), and 1 µg of mRNA was used for reverse transcription with the Superscript II reverse transcriptase (Thermo Fisher). Quantitative real-time PCR reactions were performed using a SYBR Select Master Mix and a QuantStudio 6 pro Real-Time PCR System (Thermo Fisher Scientific). Relative mRNA expression was quantified using the DDcycle threshold method and normalized to *Rpl32* as a housekeeping gene. All primers were purchased from Eurofins Genomics (Ebersberg, Germany). Primer sequences used are indicated in **Table 1**.

**Table 1:** Primers used for RT-qPCR.

Genes	Forward 5’ → 3’	Reverse 5’ → 3’
*Krt6*	CATCATTGGAGAGAGGGGTCG	CGAATTCATTCTCTGCTGCTGTG
*Krt10*	AACTGACAATGCCAACGTGC	TAGGTAGGCCAGCTCTTCGT
*Krt16*	TGGATGGCGAGAATATCCACAG	GCTCCTTGAGGATGGACCG
*Loricrin*	AGCATCACCTCCTTCCCTCA	TGGGCTGCTTTTTCTGGTGA
*Ivl*	CCTGTGAGTTTGTTTGGTCTACA	GCTGGATATGATCTGGAGAA
*Fig*	AGGACAACTACAGGCAGTCT	TTTTGCCAGCTTTAGCACCAG
*Cdsn*	TTGCTGATGGCCGGTCTTAT	GAGACAAGGGTCATTCGGGG
*Cxcl1*	ACTCAAGAATGGTCGCGAGG	GTGCCATCAGAGCAGTCTGT
*Tnfa*	AGTTCTATGGCCCAGACCCT	GTCTTTGAGATCCATGCCGT
*Il6*	TAGTCCTTCCTACCCCAATTTCC	TTGGTCCTTAGCCACTCCTTC
*Il1b*	TGTGAAATGCCACCTTTTGA	GTGCTCATGTCCTCATCCTG
*Il23a*	CCAGCGGGACATATGAATCTACT	CTTGTGGGTCACAACCATCTTC
*Il17a*	CCACGTCACCCTGGACTCTC	CTCCGCATTGACACAGCG
*Ly6g*	ATTGCAAAGTCCTGTGTGCT	TGTTGCAGGAAGTCTCAGGT

### Cytometric bead array

Skin samples of naïve and IMQ-treated mice were lysed in 500 μl TN buffer (50 mM Tris, 150 mM NaCl, complete EDTA-free protease inhibitor mixture; Roche Diagnostics, Rotkreuz, Switzerland). TNFα, IL-6, IL-1β, and CXCL1 concentrations in skin lysates and in the plasma were quantified using cytometric bead array flex sets (BD Biosciences, Franklin Lakes, NJ, USA). This cytometric bead array recognizes both the full-length proform and the mature form of IL-1β. Samples were analyzed using an LSR Fortessa flow cytometer (BD Biosciences, Franklin Lakes, NJ, USA), and the BD Biosciences FCAP software (V1.0.1). Cytokine levels in skin lysates were normalized for total protein content, assessed with the DC protein assay kit (Bio-Rad).

### Statistical analysis

Statistical significance was assessed using a Mann-Whitney test, a Kruskal-Wallis test or 2-way ANOVA, as indicated, with GraphPad Prism 10 (GraphPad Software, La Jolla, CA, USA).

## Results

### Targeted IL-38 overexpression in skin epidermis

**Figure 1:**
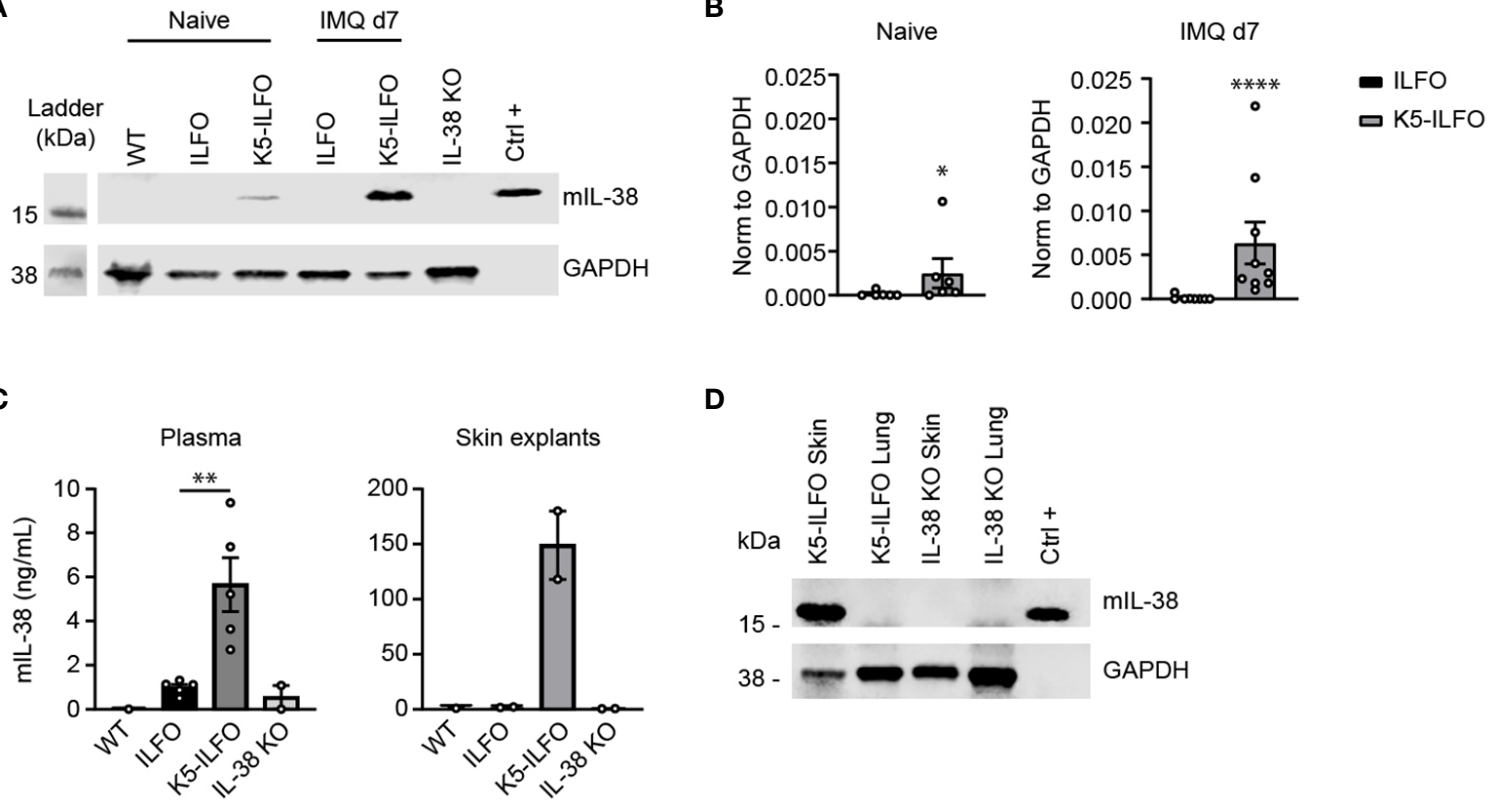
IL-38 protein overexpression in the epidermis of K5-ILFO mice. **(A)** IL-38 protein expression (upper panel) was assessed by Western blotting in the skin of naïve WT, ILFO, K5-ILFO and IL-38KO mice and of ILFO and K5-ILFO mice treated daily with 62.5 mg IMQ-containing cream on their back skin for 7 days. HEK 293T cells transfected with mIL-38 were used as a positive control (Ctrl+). Membranes were blotted also for GAPDH, used as a loading control (bottom panel). **(B)** IL-38 protein levels in the skin of naïve and IMQ-treated ILFO and K5-ILFO mice, as assessed by Western blotting, were quantified by densitometric analysis of the bands and normalized to GAPDH. Results are shown as individual values and mean ± SEM for n≥6 ILFO (black columns) and n≥6 K5-ILFO (grey columns) mice. **(C)** IL-38 concentrations were assessed by ELISA in the plasma (left panel) and in conditioned media of skin explant cultures (right panel) isolated from WT, ILFO, K5-ILFO and IL-38KO mice. Results are shown as individual values and means ± SEM for n=1-5 mice per genotype. **(D)** IL-38 protein expression was assessed by Western blotting in the skin and lung of IMQ-treated K5-ILFO mice (upper panel). Skin and lungs of naïve IL-38KO mice were used as negative controls. Membranes were also blotted for GAPDH, used as a loading control (bottom panel). *p<0.05; **p<0.01; ****p<0.0001. p-values were calculated using Mann-Whitney t-tests with FDR correction **(B, C)**.

We generated K5-ILFO mice overexpressing IL-38 in epidermal keratinocytes by breeding ILFO mice, which allow for Cre-dependent IL-38 overexpression, with K5-Cre tg mice producing the Cre recombinase under the control of the *Krt5* promoter. We confirmed specific IL-38 overexpression in the skin of K5-ILFO mice by performing Western blot analysis on skin lysates of naïve and IMQ-treated WT, ILFO and K5-ILFO mice (**Figure 1A**). Skin lysates of IL-38-deficient mice were used to validate the specificity of the detection. Overexpression of the IL-38 protein was observed specifically in the skin of K5-ILFO mice, in which IL-38 levels further increased after IMQ treatment (**Figures 1A, B**). In contrast, endogenous IL-38 protein levels in WT and ILFO mice remained below the detection limit of the Western blot. Interestingly, overexpressed IL-38 was also detected by ELISA in the plasma and in conditioned media of skin explants isolated from K5-ILFO mice (**Figure 1C**), indicating that the protein is released by keratinocytes and can be found in the circulation.

As part of the initial characterization of ILFO and K5-ILFO mice, we also investigated IL-38 expression at the mRNA level. Levels of endogenous *Il1f10* mRNA were generally low. In agreement with previous observations (5, 18), highest endogenous *Il1f10* expression was observed in the skin, where levels were similar in ILFO and K5-ILFO mice, and decreased after IMQ treatment (**Supplementary Figures S1A, C**). Using primers that amplify transgene-derived, in addition to endogenous *Il1f10* mRNA, we unexpectedly observed substantial *Ilf10* transcript levels in the skin of both ILFO and K5-ILFO mice (**Supplementary Figures S1B, C**), which was at odds with the specific IL-38 protein overexpression detected exclusively in the skin of K5-ILFO mice (**Figure 1**). Similarly, substantial *Ilf10* mRNA levels were detected in the lungs of ILFO and K5-ILFO mice, despite the absence of Cre recombinase in this mouse strain (ILFO) or organ (K5-ILFO) (**Supplementary Figure S1C**). We thus performed additional RT-PCR analyses by combining forward primers located in the Neo cassette with reverse primers binding to the IL-38 coding region. These experiments revealed the presence of bi-cistronic read-through transcripts in ILFO mice, encompassing both the Neo and the IL-38 cDNAs (data not shown). This indicates that some leakage of the transcriptional stop signal, which consists of two SV40 late polyA signals inserted after the Neo cDNA in the Neo-stop cassette, occurs *in vivo* (**Supplementary Figures S1B, C**). Production of transgene-derived IL-38 protein is however not expected in this situation, as, in eukaryotic cells, the downstream open reading frame of bi-cistronic transcripts is not efficiently translated. Accordingly, we did not detect IL-38 protein overexpression in ILFO mice (**Figure 1**), nor in the lungs of K5-ILFO mice (**Figure 1D**). Finally, as expected, excision of the Neo-stop cassette by Cre recombinase in the skin of K5-ILFO mice led to an increase in *Il1f10* mRNA expression, as compared to read-through levels (**Supplementary Figure S1C**). Altogether, these data document tissue-specific overexpression of the IL-38 protein in the epidermis of K5-ILFO mice.

### Reduced scaling during IMQ-induced skin inflammation in female K5-ILFO mice

**Figure 2:**
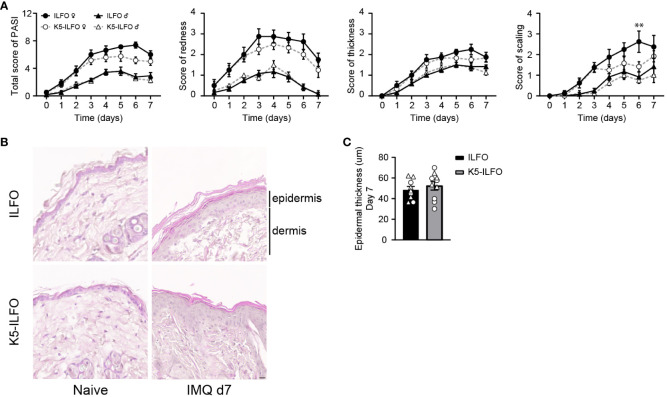
IL-38 overexpression in the epidermis reduces desquamation in female mice during IMQ-induced skin inflammation. Male and female ILFO and K5-ILFO mice were treated daily with 62.5 mg IMQ-containing cream on their back skin for 7 days. **(A)** Cumulative Psoriasis Area Severity Index (PASI) scores (left panel) were calculated daily based on the individual scores for skin redness (middle left panel), scaling (middle right panel), and stiffness (right panel) until day 7 after initial IMQ application. Data are shown as mean ± SEM for ILFO (open symbols) and K5-ILFO (black symbols) mice. Data for males (triangles, n=6 ILFO and n=4 K5-ILFO mice) and females (circles, n=4 ILFO and n=6 K5-ILFO mice) are plotted separately. **(B)** Representative pictures of H&E-stained skin sections from naïve and day 7 IMQ-treated female ILFO and K5-ILFO mice. **(C)** Epidermal thickness measurements (in µm) on day 7 in IMQ-treated male (triangles) and female (circles) ILFO (black column) and K5-ILFO (grey column) mice are shown as individual values and means ± SEM for n=10 animals per group. **p<0.01 female ILFO vs. female K5-ILFO mice, as assessed using a 2-way ANOVA with Sidak correction **(A)**.

IL-38 was previously reported to have anti-inflammatory properties in the IMQ-induced mouse model of psoriasis (7, 11). We thus examined a potential effect of epidermal IL-38 overexpression in this model by comparing K5-ILFO mice to their littermate ILFO controls. Interestingly, irrespective of the genotype, we observed significantly higher disease development in female than in male mice, as indicated by higher PASI scores (p<0.05, K5-ILFO and ILFO female vs. male mice on days 3, 6 and 7) (**Figure 2A**). This difference was mostly explained by higher scores for redness and scaling, but not for epidermal thickening, in female mice.

Contrary to our expectations, we did not observe any differences in total PASI, or skin redness or thickness scores between K5-ILFO and ILFO mice, neither in males nor in females (**Figure 2A**). Histological analysis on H&E-stained skin sections (**Figure 2B**) confirmed similar epidermal thickness in K5-ILFO and ILFO mice on day 7 (**Figure 2C**). In contrast, scaling was significantly reduced in female K5-ILFO mice, as compared to female ILFO mice on day 6 (**Figure 2A**).

### Activation and differentiation of the epidermis are not impacted by epidermal IL-38 overexpression

**Figure 3:**
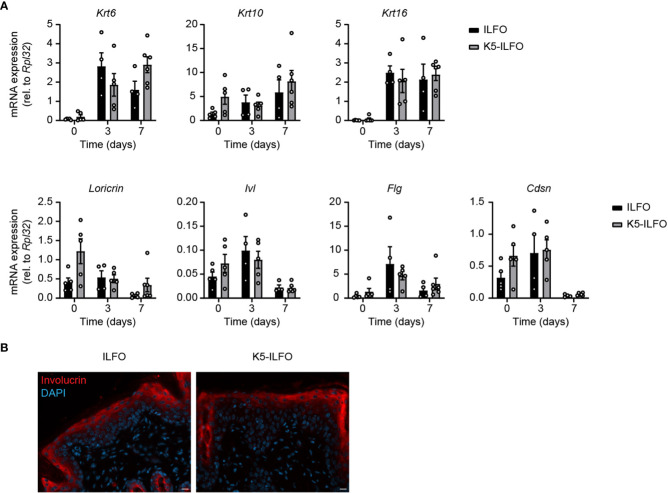
Activation and differentiation of the epidermis is not affected by epidermal IL-38 overexpression during IMQ-induced psoriasis. Female ILFO and K5-ILFO mice were treated daily with 62.5 mg IMQ-containing cream on their back skin for 3 and 7 days. **(A)** *Krt* (keratin)*6*, *Krt10*, *Krt16*, *Loricrin*, *Ivl* (involucrin), *Fig* (fillagrin) and *Cdsn* (corneodesmosin) mRNA expression was quantified by RT-qPCR in the skin of naïve male and female ILFO and K5-ILFO mice (day 0) and in IMQ-treated skin of female IFO and K5-ILFO mice on days 3 and 7. Results are shown as individual values and mean ± SEM for n≥4 ILFO (black columns) and n≥5 K5-ILFO (grey columns) mice. Significant differences in gene expression observed between naïve and IMQ-treated skin were: day 0 vs. day 3, *Krt6* (ILFO) and *Krt16* (ILFO and K5-ILFO); day 0 vs. day 7, *Krt6* (K5-ILFO), *Krt16* (ILFO and K5-ILFO), *Loricrin*, *Ivl* and *Cdsn* (K5-ILFO); p<0.05, as assessed using a Kruskal-Wallis test. **(B)** Representative picture of day 7 IMQ-treated skin of ILFO and K5-ILFO mice, stained for involucrin (red) and DAPI (blue). Scale bar 20 µm. Original magnification 20x.

We next investigated potential effects of epidermal IL-38 overexpression on keratinocyte activation and differentiation in naïve and IMQ-treated skin. Expression of mRNA encoding the activation markers keratin 6 and keratin 16 increased with IMQ treatment in all mice (**Figure 3A**), as expected (19, 20). We did not observe any difference in *Krt6* or *Krt16* levels between ILFO and K5-ILFO mice.

Expression of the keratinocyte differentiation markers *Krt10* and *Flg* remained unchanged with IMQ treatment, while *Loricrin, Ivl* and *Cdsn* mRNA levels appeared slightly reduced on day 7 (**Figure 3A**). We did not observe any significant differences in the expression of keratinocyte differentiation markers between ILFO and K5-ILFO mice. Moreover, we could not detect differences in keratin 6 or 10 expression at the protein level by Western blotting (data not shown) and immunofluorescence revealed a similar pattern of involucrin expression in IMQ-treated ILFO and K5-ILFO mice (**Figure 3B**).

### Selective effects of epidermal IL-38 overexpression on the production of inflammatory mediators in IMQ-treated mice

**Figure 4:**
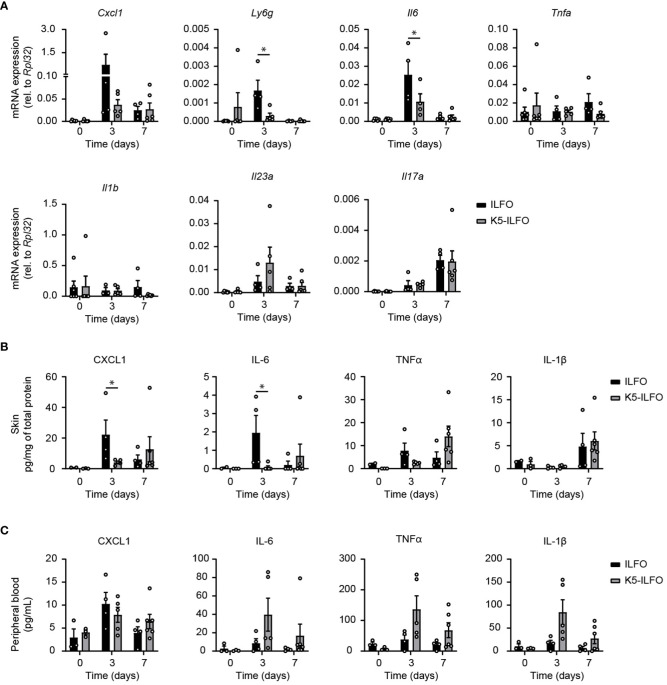
Selective effects of epidermal IL-38 overexpression on inflammatory mediator production during IMQ-induced psoriasis. Female ILFO and K5-ILFO mice were treated daily with 62.5 mg IMQ-containing cream on their back skin for 3 and 7 days. **(A)** *Cxcl1*, *Ly6g*, *Tnfa*, *Il6*, *Il1b*, *Il23a* and *IL17a* mRNA expression was quantified by RT-qPCR in the skin of naïve male and female ILFO and K5-ILFO mice (day 0) and in IMQ-treated skin of female IFO and K5-ILFO mice on days 3 and 7. Significant differences in gene expression observed between naïve and IMQ-treated skin were: day 0 vs. day 3, *Ly6g* and *Il6* (ILFO), *Cxcl1* and *Il23a* (K5-ILFO); day 0 vs. day 7, *Il17a* (ILFO and K5-ILFO); p<0.05, as assessed using a Kruskal-Wallis test. **(B)** Cytokine levels, normalized on the total protein concentration, were determined using a cytometric bead array in the skin of naïve male and female ILFO and K5-ILFO mice (day 0) and in IMQ-treated skin of female IFO and K5-ILFO mice on days 3 and 7. Significant differences in cytokine levels observed between naïve and IMQ-treated skin were: day 0 vs. day 3, CXCL1 (ILFO); day 0 vs. day 7, TNFα (K5-ILFO); p<0.05, as assessed using a Kruskal-Wallis test. **(C)** Cytokine levels were determined using a cytometric bead array in the plasma of naïve male and female ILFO and K5-ILFO mice (day 0) and of IMQ-treated female IFO and K5-ILFO mice on days 3 and 7. A significant difference in circulating TNFα levels was observed between naïve and IMQ-treated K5-ILFO mice on day 0 vs. day 3; p<0.05, as assessed using a Kruskal-Wallis test. Results are shown as individual values and means ± SEM for n≥3 ILFO (black columns) and n≥3 K5-ILFO (grey columns) mice. *p< 0.05. p-values were calculated using Mann-Whitney t-tests with FDR correction.

Finally, we examined a potential impact of epidermal IL-38 overexpression on the production of inflammatory mediators in response to IMQ treatment. The induction of CXCL1 and IL-6 mRNA and/or protein production observed after 3 days of IMQ treatment was reduced in the skin of K5-ILFO, as compared to ILFO mice (**Figures 4A, B**). Levels of mRNA encoding the neutrophil marker Ly6G were transiently increased in ILFO mice on day 3 (**Figure 4A**), which corresponds to the peak of neutrophil infiltration in this model (21). Interestingly, this increase was significantly attenuated in K5-ILFO mice. In contrast, the expression of TNFα, IL-1β, IL-23 and IL-17A mRNA and/or protein was similar in both genotypes.

At the systemic level, no significant differences in plasma CXCL1, IL-6, TNFα and IL-1β were observed between K5-ILFO and ILFO mice, although circulating IL-6, TNFα and IL-1β levels tended to be higher in the former (**Figure 4C**).

## Discussion

The IL-1 cytokine family member IL-38, which is expressed mainly in epithelia, and more particularly in keratinocytes, has been the focus of recent studies in the context of inflammatory skin diseases. In mice, the role of IL-38 was mostly studied in a model of IMQ-induced skin inflammation (7, 11, 18). IL-38 was shown to regulate keratinocyte differentiation and was proposed as an anti-inflammatory mediator during psoriasis, preventing the induction of IL-36γ-mediated pro-inflammatory responses in human keratinocytes *in vitro* and reducing the severity of inflammation, when injected into mouse skin *in vivo* (7, 11).

In the present study, we generated a genetically modified mouse strain allowing for cell-specific overexpression of IL-38, which we used to study a potential effect of IL-38 overexpression in the epidermis during IMQ-induced psoriasis. In this new mouse strain, we were able to detect the mouse IL-38 protein in the skin by Western-blotting, as well as in plasma and in conditioned media of skin explants by ELISA. Detection of the IL-38 protein in mouse tissues has proven difficult, as most commercially available antibodies show low sensitivity and generate non-specific signals, which are detected also in IL-38 KO mice, in Western blotting, immunostaining or ELISA ( (22) and AH, AW, GP unpublished results). The antibody used in this study for Western blotting was specific, but not sensitive enough to detect endogenous IL-38 levels in WT skin. In fact, in naïve K5-ILFO skin, in which *Il1f10* mRNA levels are already substantial (**Supplementary Figure S1C**), IL-38 protein expression was at the lower detection limit by Western blot. IL-38 protein expression was stronger in K5-ILFO mice after treatment with IMQ, in accordance with higher mRNA levels. IMQ treatment induces epidermal thickening, which reflects an increase in the number of keratinocyte layers, and thus in the number of keratinocytes per skin area, as a result of keratinocyte hyperproliferation (16). Since IL-38 is specifically overexpressed in keratinocytes, this increase in the relative amount of IL-38 overexpressing cells might explain the higher IL-38 mRNA and protein levels observed in IMQ-treated skin. Finally, as for Western blotting, the sensitivity of IL-38 detection by ELISA was rather modest and limited in the low nanomolar range (below 1 ng/ml in plasma, resp. 0.5 ng/ml in explant conditioned media) by non-specific signal that was present also in the IL-38 KO samples included as negative controls.

Overall, IL-38 was mainly described as an anti-inflammatory mediator exerting extracellular effects on both immune and non-immune cells (12). However, endogenous sources of extracellular IL-38 in the skin had not been defined. In particular, it was unclear whether keratinocytes, which are the main IL-38 expressing cells in this tissue, also release the protein into the extracellular space *in vivo*. Indeed IL-38, like most IL-1 family cytokines, lacks a signal peptide for conventional ER-Golgi mediated secretion and mechanisms for its release or secretion are currently unknown. We previously observed that, in cultured human keratinocytes, although IL-38 was mainly (>95%) cell-associated, a minor fraction of the protein was released into culture supernatants (8, 9). Here, we show that the IL-38 protein is specifically detected in the plasma and released from skin explants of K5-ILFO mice, thereby establishing IL-38 overexpressing keratinocytes as a source of extracellular IL-38, both in the skin and in the circulation. In addition, the overexpressed IL-38 protein likely also accumulates intracellularly in keratinocytes of K5-ILFO mice, as observed for both endogenous and overexpressed IL-38 in human keratinocytes *in vitro*, as well as for the endogenous protein in normal human skin (8, 9).

Based on previous results obtained upon injection of recombinant IL-38 protein (7, 11), we expected reduced development of IMQ-induced psoriasis in K5-ILFO mice. However, the overall clinical severity of psoriasis development was not affected by overexpression of IL-38 in keratinocytes. Similarly, there was no global effect on local and systemic inflammatory responses, in line with previous data indicating that complete ablation of IL-38 in mice delayed the resolution of IMQ-induced psoriasis, but did not modify its induction (7, 18). In particular, the IL-23/IL-17A axis that was proposed to be pathological in the IMQ model (23) was not affected by epidermal IL-38 overexpression. Instead, we observed a selective inhibition of CXCL1 and IL-6 production, as well as lower *Ly6g* expression, suggesting reduced neutrophil infiltration, in response to IMQ in the skin of K5-ILFO mice.

Based on *in vitro* results obtained using truncated recombinant proteins, IL-38 was suggested to require proteolytic N-terminal processing for full bioactivity (reviewed in 12). To date, specific cleavage site(s) or protease(s) involved have not been identified, a consensus identifying a unique, optimally active, truncated IL-38 variant has not yet emerged, and the occurrence and importance of IL-38 processing *in vivo* are unknown. Nevertheless, differential processing of the IL-38 protein by different producing cell types might affect its biological activity and thus contribute to specific biological features observed in our study for IL-38 produced by keratinocytes.

Overexpression of IL-38 in keratinocytes also affected the desquamation process during IMQ-induced psoriasis, as illustrated by reduced plaque formation on the back of female K5-ILFO mice. In line with this observation, enhanced scaling was predominant in mice lacking IL-38 when compared to enhanced inflammation (7), suggesting IL-38 as a potential regulator of desquamation in psoriasis. In contrast, we did not observe any clear effect of epidermal IL-38 overexpression on the mRNA and protein expression of several keratinocyte activation and differentiation markers. A recent study showed that excessive IL-36 receptor activation leads to a decrease in the expression of genes encoding epidermal cornified envelope-related proteins, such as corneodesmosin, and thereby enhances *stratum corneum* detachment (24). Since IL-38 was proposed to act as an IL-36 receptor antagonist (25), we explored this potential mechanism. We did however not observe any significant difference in *Cdsn* expression between IL-38 overexpressing or control mice. In addition to expression levels of cornified layer-related genes, post-translational processes, such as tightly controlled proteolysis, play an essential role in regulating skin desquamation (26). A potential role of IL-38 in modulating additional cellular processes leading to plaque formation thus remains to be investigated.

In any case, the observed selective and sex-dependent effect of epidermal IL-38 overexpression on specific disease-associated readouts suggests a more complex role of IL-38 in the inflamed skin than previously recognized. The use of ILFO mice for targeted IL-38 overexpression in different cell types, for example in the immune compartment, will help to improve our understanding of the function of IL-38, derived from different sources, in the regulation of epidermal homeostasis and inflammatory responses in the skin.

Finally, this study revealed marked differences in psoriasis development between male and female mice, mostly due to stronger erythema in females. Although many studies have used this model of IMQ-induced skin inflammation, only few compared male and female mice (27). Previous comparisons highlighted increased epidermal thickening, stronger induction of inflammatory genes, and more severe systemic effects in female than in male mice (28, 29). IMQ is an agonist for TLR7 (30) and the *TLR7* gene is located on the X chromosome and was reported to escape X chromosome inactivation in immune cells, at least in humans (31). Increased TLR7 gene dosage might thus contribute to a stronger response to IMQ in female mice.

Taken together, our results validate the generation of a new mouse line allowing for tissue-specific overexpression of IL-38. These mice will be a useful tool for future studies of the biology of IL-38 in the skin, but also in other organs, by breeding ILFO mice to cell-type specific Cre strains. Moreover, the ability to detect the IL-38 protein *in vivo* in these mice will enable us to examine the production and potential release of this protein by different cell types and clarify its various functions. Finally, this study highlights a potential role of IL-38 in the regulation of skin desquamation.

## Data availability statement

The data presented in the study are deposited in the Yareta repository (https://doi.org/10.26037/yareta:d47b2igrzbgfhghgd6n543f2e4).

## Ethics statement

The animal study was approved by the Geneva Veterinarian Office (Service de la consommation et des affaires vétérinaires, authorization GE161). The study was conducted in accordance with the local legislation and institutional requirements.

## Author contributions

AH: Conceptualization, Methodology, Project administration, Validation, Writing – original draft, Writing – review & editing, Formal analysis, Investigation, Visualization. ER: Investigation, Methodology, Writing – original draft, Writing – review & editing. DT-A: Writing – original draft, Writing – review & editing, Investigation, Methodology, Validation. AW: Methodology, Writing – original draft, Writing – review & editing. GP: Conceptualization, Funding acquisition, Methodology, Project administration, Supervision, Validation, Writing – original draft, Writing – review & editing.
