# Supplementary material for: Interleukin-38 overexpression in keratinocytes limits desquamation but does not affect the global severity of imiquimod-induced skin inflammation in mice
Source: Front Immunol. 2024 Jul 25;15:1387921. doi: 10.3389/fimmu.2024.1387921 (PMC11306934; doi:10.3389/fimmu.2024.1387921)
Supplement: Supplementary file 1 [file DataSheet_1.pdf]

Supplementary Figure

Figure S1. Generation and characterization of ILFO and K5-ILFO mice

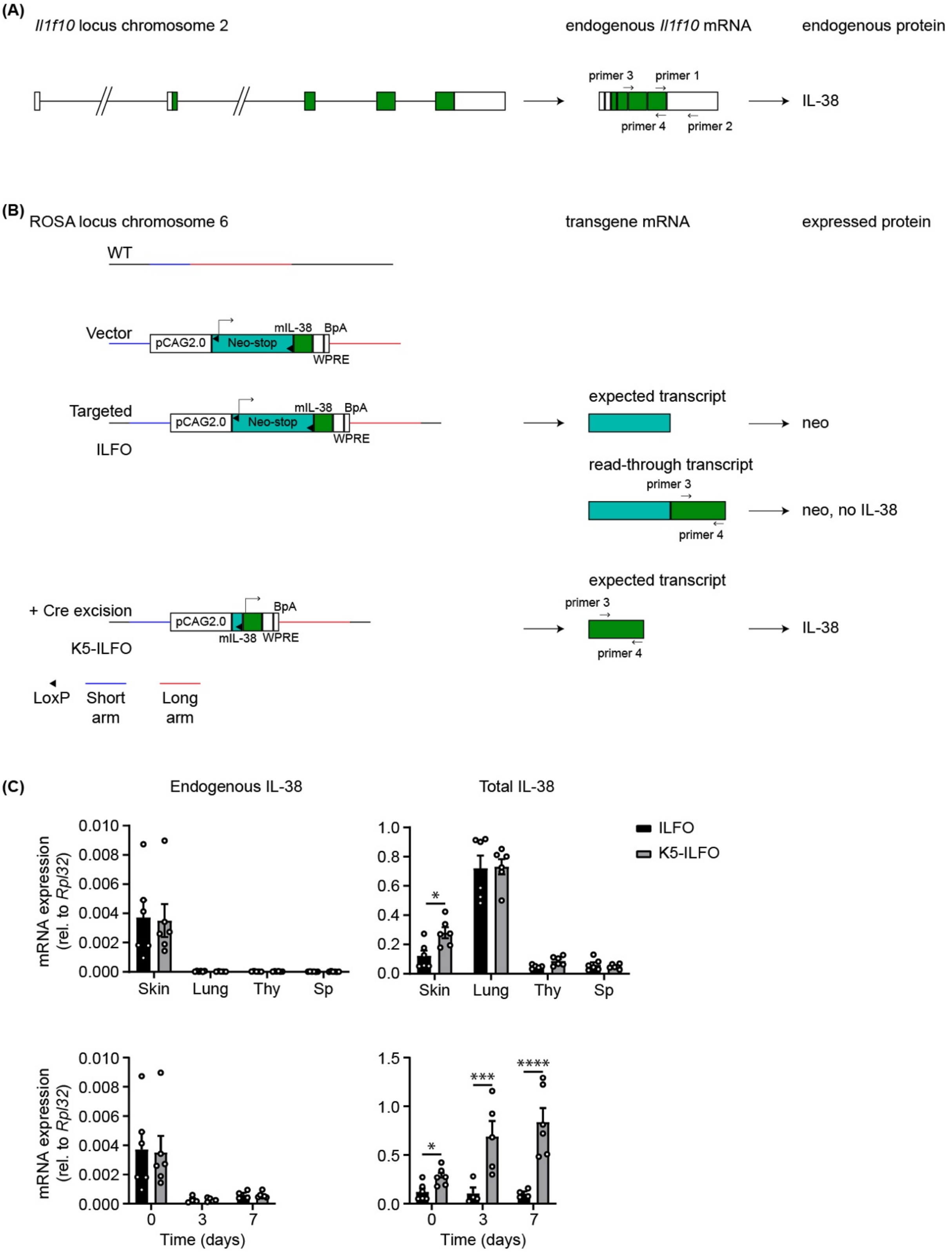

(A) Schematic representation of the mouse *Il1f10* gene on chromosome 2 (left) and of the endogenous *Il1f10* mRNA (centre) encoding the IL-38 protein. (B) Schematic representation of (left, from top to bottom): the wild-type (WT) ROSA locus on chromosome 6, the targeting vector used for the generation of ILFO mice, the resulting targeted locus in ILFO mice, and the Cre-excised locus in Cre-expressing cells of K5-ILFO mice. The targeting construct is based on a ROSA26 backbone vector containing a pCAG promoter and a floxed Neo-stop cassette. The Neo cassette is followed by the *Il1f10* cDNA encoding mouse IL-38 (mIL-38), a WPRE sequence and a BGH polyA (BpA) signal. The short homology arm (SA, blue line) corresponds to 1.1 kb of ROSA26 genomic sequence and the long arm (LA, red line) to 4.3 kb. LoxP sites are represented by triangles. Expected and read-through transcripts (centre), as well as corresponding protein products (right) are indicated for each allele. (A and B) The Neo-stop cassette is shown in blue and the IL-38 coding sequence in green. Non-coding sequences are symbolized by open boxes. The location of primers used for the detection of endogenous (primers 1 and 2) and total (endogenous and transgenic expressed from the ILFO cassette; primers 3 and 4) *Il1f10* transcripts are indicated. Transcripts and primers are not drawn to scale. (C) Expression of endogenous (primers 1+2, left panels) and total (primers 3+4, right panels) levels of mRNA encoding IL-38 was assessed by RT-qPCR in skin, lung, thymus (Thy) and spleen (Sp) of naïve ILFO (black columns) and K5-ILFO (grey columns) mice (top panels) and on day 0, day 3 and day 7 following IMQ-treatment in the skin of ILFO (black columns) and K5-ILFO (grey columns) mice (bottom panels). Results are shown as individual values and means $\pm$ SEM for $n \geq 4$ mice. ***$p<0.001$, ****$p<0.0001$, as assessed using Mann-Whitney t-tests with FDR correction.
